# Poly[[bis(μ_2_-4-amino­benzene­sul­fon­ato-κ^2^ *N*:*O*)diaqua­manganese(II)] dihydrate]

**DOI:** 10.1107/S1600536808025579

**Published:** 2008-08-13

**Authors:** Zhan Ling Li, Ya Wen Xuan, Wen Wu, Dong Po Xie

**Affiliations:** a Department of Chemistry, Zhoukou Normal University, Zhoukou 466001, People’s Republic of China

## Abstract

The title compound, {[Mn(NH_2_C_6_H_4_SO_3_)_2_(H_2_O)_2_]·2H_2_O}_*n*_, was prepared under mild hydro­thermal conditions. The unique Mn^II^ ion is located on a crystallographic inversion center and is coordinated by two –NH_2_ and two –SO_3_ groups from four 4-amino­benzene­sulfonate ligands and by two water mol­ecules in the axial positions, forming a slightly distorted octa­hedral coordination environment. The 4-amino­benzene­sulfonate anions behave as μ_2_-bridging ligands to produce a two-dimensional structure. In the crystal structure, inter­molecular N—H⋯O, O—H⋯O and C—H⋯O hydrogen bonds link the layers into a three-dimensional network.

## Related literature

For the isostructural Zn and Co compounds, see: Shakeri & Haussuhl (1992 ▶). For a similar layered structure, see: Cai *et al.* (2003 ▶).
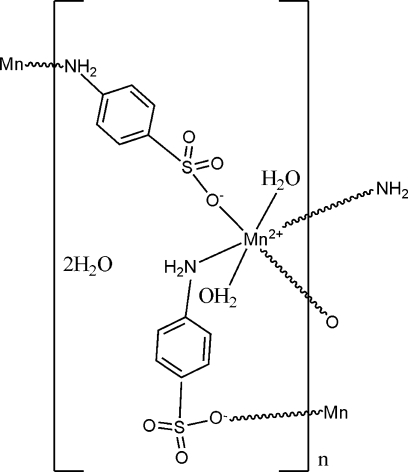


## Experimental

### 

#### Crystal data

[Mn(C_6_H_6_NO_3_S)_2_(H_2_O)_2_]·2H_2_O
*M* *_r_* = 471.36
Monoclinic, 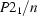

*a* = 7.4485 (8) Å
*b* = 17.4102 (19) Å
*c* = 7.6509 (9) Å
β = 116.688 (1)°
*V* = 886.47 (17) Å^3^
*Z* = 2
Mo *K*α radiation
μ = 1.04 mm^−1^
*T* = 295 (2) K
0.49 × 0.45 × 0.45 mm

#### Data collection

Bruker SMART CCD diffractometer
Absorption correction: multi-scan (*SADABS*; Bruker, 2002 ▶) *T*_min_ = 0.547, *T*_max_ = 0.625
6604 measured reflections
1637 independent reflections
1585 reflections with *I* > 2σ(*I*)
*R*_int_ = 0.015

#### Refinement

*R*[*F* ^2^ > 2σ(*F* ^2^)] = 0.048
*wR*(*F* ^2^) = 0.149
*S* = 1.11
1637 reflections
124 parameters
H-atom parameters constrained
Δρ_max_ = 1.19 e Å^−3^
Δρ_min_ = −1.03 e Å^−3^

### 

Data collection: *SMART* (Bruker, 2002 ▶); cell refinement: *SAINT* (Bruker, 2002 ▶); data reduction: *SAINT*; program(s) used to solve structure: *SHELXS97* (Sheldrick, 2008 ▶); program(s) used to refine structure: *SHELXL97* (Sheldrick, 2008 ▶); molecular graphics: *SHELXTL* (Sheldrick, 2008 ▶); software used to prepare material for publication: *SHELXTL*.

## Supplementary Material

Crystal structure: contains datablocks I, global. DOI: 10.1107/S1600536808025579/lh2671sup1.cif

Structure factors: contains datablocks I. DOI: 10.1107/S1600536808025579/lh2671Isup2.hkl

Additional supplementary materials:  crystallographic information; 3D view; checkCIF report

## Figures and Tables

**Table:** 

Mn1—O4	1.993 (3)
Mn1—N1^i^	2.058 (3)
Mn1—O1	2.425 (3)

**Table:** 

O4—Mn1—O4^ii^	180
O4—Mn1—N1^i^	92.95 (13)
O4—Mn1—N1^iii^	87.05 (13)
N1^i^—Mn1—N1^iii^	180
O4—Mn1—O1^ii^	84.94 (12)
O4—Mn1—O1	95.06 (12)
N1^i^—Mn1—O1	86.66 (11)
N1^iii^—Mn1—O1	93.34 (11)
O1^ii^—Mn1—O1	180

Symmetry codes: (i) 
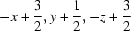
; (ii) 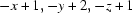; (iii) 
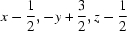
.

**Table 2:** Hydrogen-bond geometry (Å, °)

*D*—H⋯*A*	*D*—H	H⋯*A*	*D*⋯*A*	*D*—H⋯*A*
N1—H1*B*⋯O2^iv^	0.90	2.46	2.980 (4)	117
O5—H3*W*⋯O1	0.82	2.06	2.855 (5)	164
C2—H2⋯O2	0.93	2.54	2.920 (5)	105
N1—H1*B*⋯O2^iii^	0.90	2.41	3.217 (4)	149
O4—H2*W*⋯O5^v^	0.83	1.83	2.651 (5)	175
C2—H2⋯O5^v^	0.93	2.53	3.431 (6)	164
O4—H1*W*⋯O3^vi^	0.82	2.02	2.795 (4)	157
N1—H1*A*⋯O3^vii^	0.90	2.24	3.070 (5)	153
C3—H3⋯O3^vii^	0.93	2.55	3.300 (5)	138
O5—H4*W*⋯O2^viii^	0.82	2.00	2.815 (5)	175

Symmetry codes: (iii) 
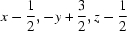
; (iv) 
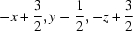
; (v) 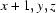; (vi) 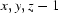; (vii) 
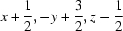
; (viii) 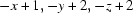.

**Fig. 1.**
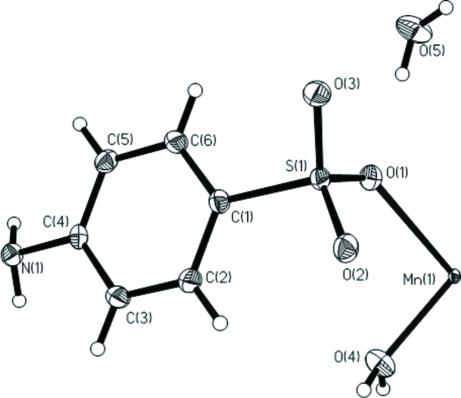
The asymmetric unit of the title compound showing 30% probability ellipsoids.

**Fig. 2.**
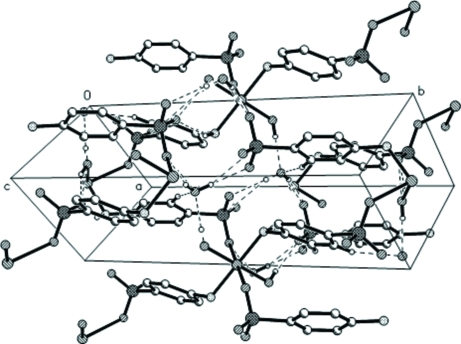
Part of the crystal structure of the title compound showing hydrogen bonds as dashed lines.

## supplementary crystallographic information

### Comment

The asymmetric unit of the title compound (I) is illustrated in Fig.1. This consists of one half of Mn^II^ ion, one 4-aminobenzenesulfonate ligand, one coordinated water molecule and one solvent water molecule. The title compound is isostructural with the Cobalt and Zinc analogs (Shakeri & Haussuhl, 1992). It is interesting to note that the title compound has very similar layered structure as that observed in [Cd(1,5 nds)-(H_2_O)_2_]_n_ (Cai *et al.*,  2003) (1,5-nds = 1,5-naphthalenedisulfonate) in which the Cd^II^ ion is also coordinated octahedrally by two water molecules occupying the axial positions and the layers are connected by hydrogen bonds formed between the coordinated water molecules and the sulfonate O atoms. In the crystal structure of (I) inter-layered hydrogen bonds formed between the coordinated water molecules and the –NH_2_ groups with the free –SO_3_^-^ oxygen atoms generate an extended 3-D structure (Fig.2)

### Experimental

All the reagents were of AR grade and used without further purification. *p*-anilinesulfonic acid (0.8690 g, 5 mmol) were dissolved in 50 ml H_2_O solution, the mixed solution was basified with 1 mol.*L*^-1^ KOH to pH =7.5. Then the resultant solution was added in 10 ml double-distilled water containing MnCl_2_.4H_2_O (0.3950 g, 2 mmol), the resulting solution was heated at 423 K for 96 h. After cooling to room temperature, block crystals were obtained in a yield up to 37.6%.

### Refinement

H atoms bonded to O atoms were included in 'as found' positions and refined with *U*_iso_(H)=1.5U_eq_(O). Other H atoms were positioned geometrically and refined using a riding model, with C-H = 0.97 Å ; N-H = 0.90 Å and with *U*_iso_(H)=1.2 times *U*_eq_(C,N).

### Crystal data

**Table:**

[Mn(C_6_H_6_NO_3_S)_2_(H_2_O)_2_]·2H_2_O	*F*_000_ = 486
*M**_r_* = 471.36	*D*_x_ = 1.766 Mg m^−^^3^
Monoclinic, *P*2_1_/*n*	Mo *K*α radiation λ = 0.71073 Å
Hall symbol: -P 2yn	Cell parameters from 2041 reflections
*a* = 7.4485 (8) Å	θ = 2.5–26.2º
*b* = 17.4102 (19) Å	µ = 1.04 mm^−^^1^
*c* = 7.6509 (9) Å	*T* = 295 (2) K
β = 116.688 (1)º	Block, yellow
*V* = 886.47 (17) Å^3^	0.49 × 0.45 × 0.45 mm
*Z* = 2	

### Data collection

**Table:**

Bruker SMART CCD diffractometer	1637 independent reflections
Radiation source: fine-focus sealed tube	1585 reflections with *I* > 2σ(*I*)
Monochromator: graphite	*R*_int_ = 0.015
Detector resolution: 0 pixels mm^-1^	θ_max_ = 25.5º
*T* = 295(2) K	θ_min_ = 2.3º
φ and ω scans	*h* = −9→9
Absorption correction: multi-scan(SADABS; Bruker, 2002)	*k* = −19→20
*T*_min_ = 0.547, *T*_max_ = 0.625	*l* = −9→9
6604 measured reflections	

### Refinement

**Table:**

Refinement on *F*^2^	Secondary atom site location: difference Fourier map
Least-squares matrix: full	Hydrogen site location: inferred from neighbouring sites
*R*[*F*^2^ > 2σ(*F*^2^)] = 0.048	H-atom parameters constrained
*wR*(*F*^2^) = 0.149	*w* = 1/[σ^2^(*F*_o_^2^) + (0.0902*P*)^2^ + 2.4519*P*] where *P* = (*F*_o_^2^ + 2*F*_c_^2^)/3
*S* = 1.11	(Δ/σ)_max_ < 0.001
1637 reflections	Δρ_max_ = 1.19 e Å^−^^3^
124 parameters	Δρ_min_ = −1.03 e Å^−^^3^
Primary atom site location: structure-invariant direct methods	Extinction correction: none

### Special details

**Table:**

Geometry. All e.s.d.'s (except the e.s.d. in the dihedral angle between two l.s. planes) are estimated using the full covariance matrix. The cell e.s.d.'s are taken into account individually in the estimation of e.s.d.'s in distances, angles and torsion angles; correlations between e.s.d.'s in cell parameters are only used when they are defined by crystal symmetry. An approximate (isotropic) treatment of cell e.s.d.'s is used for estimating e.s.d.'s involving l.s. planes.
Refinement. Refinement of *F*^2^ against ALL reflections. The weighted *R*-factor *wR* and goodness of fit *S* are based on *F*^2^, conventional *R*-factors *R* are based on *F*, with *F* set to zero for negative *F*^2^. The threshold expression of *F*^2^ > σ(*F*^2^) is used only for calculating *R*-factors(gt) *etc*. and is not relevant to the choice of reflections for refinement. *R*-factors based on *F*^2^ are statistically about twice as large as those based on *F*, and *R*- factors based on ALL data will be even larger.

### Fractional atomic coordinates and isotropic or equivalent isotropic displacement parameters (Å^2^)

**Table:**

	*x*	*y*	*z*	*U*_iso_*/*U*_eq_	
Mn1	0.5000	1.0000	0.5000	0.0103 (3)	
S1	0.65941 (14)	0.89771 (5)	0.94564 (13)	0.0222 (3)	
O1	0.4931 (4)	0.92302 (17)	0.7611 (4)	0.0299 (7)	
O2	0.8422 (4)	0.94184 (16)	0.9916 (4)	0.0315 (7)	
O3	0.6018 (5)	0.89731 (17)	1.1046 (4)	0.0333 (7)	
O4	0.7291 (5)	0.9436 (2)	0.4921 (4)	0.0376 (8)	
H1W	0.7114	0.9406	0.3785	0.056*	
H2W	0.8495	0.9411	0.5706	0.056*	
N1	0.8133 (5)	0.57368 (19)	0.7847 (5)	0.0265 (7)	
H1A	0.8601	0.5771	0.6950	0.032*	
H1B	0.6918	0.5512	0.7253	0.032*	
C1	0.7134 (6)	0.8009 (2)	0.9110 (5)	0.0238 (8)	
C2	0.8724 (6)	0.7854 (2)	0.8697 (6)	0.0307 (9)	
H2	0.9547	0.8250	0.8669	0.037*	
C3	0.9088 (6)	0.7106 (2)	0.8325 (6)	0.0309 (9)	
H3	1.0167	0.6997	0.8065	0.037*	
C4	0.7828 (6)	0.6515 (2)	0.8344 (5)	0.0236 (8)	
C5	0.6261 (6)	0.6673 (2)	0.8804 (6)	0.0289 (9)	
H5	0.5452	0.6276	0.8857	0.035*	
C6	0.5900 (6)	0.7421 (2)	0.9184 (6)	0.0288 (9)	
H6	0.4847	0.7529	0.9485	0.035*	
O5	0.1093 (5)	0.9329 (2)	0.7587 (5)	0.0484 (9)	
H3W	0.2066	0.9285	0.7362	0.073*	
H4W	0.1188	0.9679	0.8342	0.073*	

### Atomic displacement parameters (Å^2^)

**Table:**

	*U*^11^	*U*^22^	*U*^33^	*U*^12^	*U*^13^	*U*^23^
Mn1	0.0192 (4)	0.0266 (4)	0.0139 (4)	−0.0005 (2)	0.0062 (3)	−0.0015 (2)
S1	0.0264 (5)	0.0180 (5)	0.0240 (5)	0.0012 (3)	0.0129 (4)	−0.0003 (3)
O1	0.0309 (15)	0.0283 (15)	0.0302 (15)	0.0046 (12)	0.0134 (12)	0.0053 (12)
O2	0.0311 (15)	0.0227 (15)	0.0398 (16)	−0.0025 (12)	0.0153 (13)	−0.0021 (12)
O3	0.0452 (18)	0.0300 (16)	0.0328 (15)	0.0003 (13)	0.0246 (14)	−0.0025 (12)
O4	0.0328 (16)	0.050 (2)	0.0276 (15)	0.0088 (14)	0.0110 (13)	−0.0041 (14)
N1	0.0320 (18)	0.0209 (17)	0.0281 (17)	−0.0011 (13)	0.0147 (15)	−0.0042 (13)
C1	0.0269 (19)	0.0205 (18)	0.0237 (18)	0.0017 (15)	0.0110 (15)	−0.0006 (14)
C2	0.036 (2)	0.021 (2)	0.042 (2)	−0.0019 (16)	0.023 (2)	−0.0006 (17)
C3	0.031 (2)	0.027 (2)	0.042 (2)	0.0011 (17)	0.0226 (19)	−0.0010 (17)
C4	0.028 (2)	0.0185 (18)	0.0210 (18)	0.0038 (14)	0.0081 (15)	0.0015 (14)
C5	0.031 (2)	0.025 (2)	0.032 (2)	−0.0039 (16)	0.0153 (17)	0.0010 (16)
C6	0.032 (2)	0.025 (2)	0.035 (2)	0.0006 (16)	0.0199 (18)	−0.0024 (16)
O5	0.0323 (17)	0.064 (2)	0.051 (2)	−0.0065 (16)	0.0208 (16)	−0.0216 (18)

### Geometric parameters (Å, °)

**Table:**

Mn1—O4	1.993 (3)	N1—H1A	0.9000
Mn1—O4^i^	1.993 (3)	N1—H1B	0.9000
Mn1—N1^ii^	2.058 (3)	C1—C2	1.383 (6)
Mn1—N1^iii^	2.058 (3)	C1—C6	1.393 (6)
Mn1—O1^i^	2.425 (3)	C2—C3	1.385 (6)
Mn1—O1	2.425 (3)	C2—H2	0.9300
S1—O3	1.460 (3)	C3—C4	1.396 (6)
S1—O2	1.462 (3)	C3—H3	0.9300
S1—O1	1.467 (3)	C4—C5	1.390 (6)
S1—C1	1.780 (4)	C5—C6	1.387 (6)
O4—H1W	0.8200	C5—H5	0.9300
O4—H2W	0.8267	C6—H6	0.9300
N1—C4	1.453 (5)	O5—H3W	0.8197
N1—Mn1^iv^	2.058 (3)	O5—H4W	0.8216
			
O4—Mn1—O4^i^	180	C4—N1—Mn1^iv^	120.1 (2)
O4—Mn1—N1^ii^	92.95 (13)	C4—N1—H1A	107.3
O4^i^—Mn1—N1^ii^	87.05 (13)	Mn1^iv^—N1—H1A	107.3
O4—Mn1—N1^iii^	87.05 (13)	C4—N1—H1B	107.3
O4^i^—Mn1—N1^iii^	92.95 (13)	Mn1^iv^—N1—H1B	107.3
N1^ii^—Mn1—N1^iii^	180	H1A—N1—H1B	106.9
O4—Mn1—O1^i^	84.94 (12)	C2—C1—C6	121.0 (4)
O4^i^—Mn1—O1^i^	95.06 (12)	C2—C1—S1	119.5 (3)
N1^ii^—Mn1—O1^i^	93.34 (11)	C6—C1—S1	119.5 (3)
N1^iii^—Mn1—O1^i^	86.66 (11)	C1—C2—C3	119.8 (4)
O4—Mn1—O1	95.06 (12)	C1—C2—H2	120.1
O4^i^—Mn1—O1	84.94 (12)	C3—C2—H2	120.1
N1^ii^—Mn1—O1	86.66 (11)	C2—C3—C4	119.7 (4)
N1^iii^—Mn1—O1	93.34 (11)	C2—C3—H3	120.1
O1^i^—Mn1—O1	180	C4—C3—H3	120.1
O3—S1—O2	113.12 (18)	C5—C4—C3	120.1 (4)
O3—S1—O1	111.46 (18)	C5—C4—N1	119.9 (4)
O2—S1—O1	111.50 (18)	C3—C4—N1	119.9 (4)
O3—S1—C1	106.85 (18)	C6—C5—C4	120.2 (4)
O2—S1—C1	106.57 (18)	C6—C5—H5	119.9
O1—S1—C1	106.90 (18)	C4—C5—H5	119.9
S1—O1—Mn1	129.61 (17)	C5—C6—C1	119.2 (4)
Mn1—O4—H1W	109.4	C5—C6—H6	120.4
Mn1—O4—H2W	132.0	C1—C6—H6	120.4
H1W—O4—H2W	111.8	H3W—O5—H4W	114.3
			
O3—S1—O1—Mn1	143.8 (2)	C6—C1—C2—C3	0.8 (6)
O2—S1—O1—Mn1	16.3 (3)	S1—C1—C2—C3	−176.6 (3)
C1—S1—O1—Mn1	−99.8 (2)	C1—C2—C3—C4	0.9 (6)
O4—Mn1—O1—S1	45.3 (2)	C2—C3—C4—C5	−2.4 (6)
O4^i^—Mn1—O1—S1	−134.7 (2)	C2—C3—C4—N1	176.5 (4)
N1^ii^—Mn1—O1—S1	−47.3 (2)	Mn1^iv^—N1—C4—C5	−91.0 (4)
N1^iii^—Mn1—O1—S1	132.7 (2)	Mn1^iv^—N1—C4—C3	90.1 (4)
O3—S1—C1—C2	−141.2 (3)	C3—C4—C5—C6	2.1 (6)
O2—S1—C1—C2	−20.0 (4)	N1—C4—C5—C6	−176.8 (4)
O1—S1—C1—C2	99.4 (3)	C4—C5—C6—C1	−0.4 (6)
O3—S1—C1—C6	41.3 (4)	C2—C1—C6—C5	−1.1 (6)
O2—S1—C1—C6	162.5 (3)	S1—C1—C6—C5	176.4 (3)
O1—S1—C1—C6	−78.2 (4)		

Symmetry codes: (i) −*x*+1, −*y*+2, −*z*+1; (ii) −*x*+3/2, *y*+1/2, −*z*+3/2; (iii) *x*−1/2, −*y*+3/2, *z*−1/2; (iv) −*x*+3/2, *y*−1/2, −*z*+3/2.

### Hydrogen-bond geometry (Å, °)

**Table:**

*D*—H···*A*	*D*—H	H···*A*	*D*···*A*	*D*—H···*A*
N1—H1B···O2^iv^	0.90	2.46	2.980 (4)	117
O5—H3W···O1	0.82	2.06	2.855 (5)	164
C2—H2···O2	0.93	2.54	2.920 (5)	105
N1—H1B···O2^iii^	0.90	2.41	3.217 (4)	149
O4—H2W···O5^v^	0.83	1.83	2.651 (5)	175
C2—H2···O5^v^	0.93	2.53	3.431 (6)	164
O4—H1W···O3^vi^	0.82	2.02	2.795 (4)	157
N1—H1A···O3^vii^	0.90	2.24	3.070 (5)	153
C3—H3···O3^vii^	0.93	2.55	3.300 (5)	138
O5—H4W···O2^viii^	0.82	2.00	2.815 (5)	175

Symmetry codes: (iv) −*x*+3/2, *y*−1/2, −*z*+3/2; (iii) *x*−1/2, −*y*+3/2, *z*−1/2; (v) *x*+1, *y*, *z*; (vi) *x*, *y*, *z*−1; (vii) *x*+1/2, −*y*+3/2, *z*−1/2; (viii) −*x*+1, −*y*+2, −*z*+2.
